# Investigating attention‐deficit hyperactivity disorder and autism spectrum disorder traits in the general population: What happens in adult life?

**DOI:** 10.1111/jcpp.13297

**Published:** 2020-07-14

**Authors:** Lucy Riglin, Beate Leppert, Kate Langley, Ajay K. Thapar, Michael C. O'Donovan, George Davey Smith, Evie Stergiakouli, Kate Tilling, Anita Thapar

**Affiliations:** ^1^ Division of Psychological Medicine and Clinical Neurosciences MRC Centre for Neuropsychiatric Genetics and Genomics Cardiff University Cardiff UK; ^2^ MRC Integrative Epidemiology Unit University of Bristol Bristol UK; ^3^ School of Psychology Cardiff University Cardiff UK

**Keywords:** Neurodevelopmental, attention‐deficit hyperactivity disorder, autism spectrum disorder, adult, genetic, Avon Longitudinal Study of Parents and Children

## Abstract

**Background:**

Attention‐deficit hyperactivity disorder (ADHD) and autism spectrum disorder (ASD) are generally considered early‐onset disorders so most research has therefore tended to focus on children. Differences between ADHD/ASD in adult life and childhood have been noted, but few population‐based studies have examined them in adulthood. Furthermore, the interpretation of findings is hampered by changes in measure and from parent report to self‐report.

**Method:**

We examined continuous/trait measures of parent‐ and self‐rated ADHD and ASD in adulthood (age 25 years) in a UK prospective longitudinal sample ALPSAC (the Avon Longitudinal Study of Parents and Children), using many of the same measures that parents reported on in childhood (*N* = 6,064). Our aim was to investigate these traits in this population for mean‐level sex differences, overlaps with other cognitive, learning and communication problems and their associations with polygenic risk scores (PRS) for neuropsychiatric disorders (ADHD, ASD, schizophrenia, depression and anxiety).

**Results:**

ADHD and ASD traits in adulthood, as in childhood, showed associations with childhood cognitive, learning and communication problems and adult communication/language measures, although less so for self‐ratings than parent‐ratings. Males had higher ADHD and ASD trait levels, but this was not as marked as in childhood. In adulthood, ADHD (both parent‐ and self‐rated) and ASD (parent‐rated) symptoms showed associations with ADHD PRS; self‐reported ADHD also showed association with depression PRS, whereas self‐reported ASD did not show strong PRS associations.

**Conclusions:**

Our findings suggest that in young adults, ADHD and ASD symptoms have similar characteristics as they do in childhood. Associations with other cognitive, learning and communication problems, and ADHD PRS were somewhat less pronounced for self‐reported adult ADHD and ASD symptoms, suggesting that even at age 25, parent reports, where available, could be clinically useful.

## Introduction

Attention‐deficit hyperactivity disorder (ADHD) and autism spectrum disorder (ASD) are viewed in DSM‐5 as neurodevelopmental disorders (American Psychiatric Association, 2013): alongside other disorders such as intellectual disability, specific learning disorders and communication disorders. While ADHD and ASD are defined categorically for clinical purposes, in population‐based samples, total symptom scores behave as continuously distributed traits without clear‐cut thresholds in terms of associations with adverse outcomes (Rutter et al., 2011; Thapar & Cooper, 2016). Childhood ADHD and ASD traits also show associations with the same set of early environmental and genetic risks as their respective clinical disorders (Mandy & Lai, 2016; Robinson et al., 2016; Thapar & Cooper, 2016). Traditionally, because neurodevelopmental disorders have been considered as early‐life conditions, most research has focused on children. In childhood, ADHD and ASD traits are highly correlated with each other and with other DSM‐5 neurodevelopmental problems (e.g. intellectual disability, language disorders), show a male preponderance and are highly heritable (Sullivan, Daly, & O'Donovan, 2012; Thapar, Cooper, & Rutter, 2017).

More recent population‐based studies have utilised polygenic risk scores (PRS), an index of genetic liability derived from genome‐wide association study (GWAS) data for different psychiatric disorders. Such studies have observed that child ADHD and ASD traits are associated with PRS for ADHD diagnosis (Demontis et al., 2019; Serdarevic et al., 2020) and that child ASD traits also are associated with both ASD and schizophrenia PRS (St Pourcain et al., 2018). Genome‐wide association studies further show genetic correlations between ADHD and ASD diagnoses and that ADHD and ASD diagnoses genetically overlap with other psychiatric disorders, in particular depression (Demontis et al., 2019; Grove et al., 2019) and also anxiety (in the case of ADHD; Purves et al., 2019).

Although increasingly recognised that ADHD and ASD extend into adult life in many cases, population‐based research into adult ADHD and ASD is limited (Brugha et al., 2011; Brugha et al., 2012; Kessler et al., 2006; Magiati, Tay, & Howlin, 2014; Simon, Czobor, Balint, Meszaros, & Bitter, 2009; Thapar & Cooper, 2016). Some research suggests differences between these disorders in adulthood compared with childhood. First, twin studies initially observed lower heritability estimates for adult ADHD and ASD symptoms compared with those reported in childhood (Larsson et al., 2013; Polderman, Hoekstra, Posthuma, & Larsson, 2014; Reiersen, Constantino, Grimmer, Martin, & Todd, 2008). However, this may be due to differences in informant – reflecting a switch from parent‐report to self‐report, rather than due to genuine developmental differences. Consistent with this, a patient registry‐based study found similar heritability estimates for childhood and adulthood ADHD (Larsson, Chang, D'Onofrio, & Lichtenstein, 2014) and a twin study of parent‐reported ASD symptoms in both childhood and at age 18 years reported similar heritability estimates at both ages (Taylor, Gillberg, Lichtenstein, & Lundström, 2017) – although only moderate genetic stability was observed (rG = 0.49, CI = 0.70–0.81). A recent GWAS of adult ADHD diagnosis found a genetic correlation with an independent GWAS of child ADHD to be 0.81 (*SE* = 0.09), suggesting considerable genetic overlap, although this study could not distinguish childhood‐limited ADHD from ADHD that persisted into adulthood (Rovira et al., 2019).

ASD in adult life, as in childhood, appears to be more common in males than females (Brugha et al., 2011), whereas for ADHD, reported developmental differences include the observation that adult ADHD does not appear to show the same male excess (Biederman et al., 1994), or extent of cognitive deficits (Moffitt et al., 2015; Schoechlin & Engel, 2005) as childhood ADHD. However, it is not clear whether these apparent differences also could result from methodological issues including changes in informant or ADHD measure, delayed detection in females or referral bias in adult clinic‐based studies; those with the most severe neurodevelopmental disorders may be under‐represented in healthcare settings because they do not seek help in adult life, or vanish into the criminal justice rather than health system (Young et al., 2011; Young et al., 2018). Furthermore, some individuals with adult ADHD show a late onset (Asherson & Agnew‐Blais, 2019), and it is unclear whether this group differs from early‐onset cases with respect to sex ratio, or degree of cognitive deficit.

Our aim was to examine young adult ADHD and ASD at age 25 years in a UK pregnancy birth cohort, ALSPAC, using many of the same measures and informants as were used in childhood. We set out to investigate whether ADHD and ASD traits in young adulthood show similar characteristics as have been reported in childhood, by examining associations with other neurodevelopmental problems (IQ, reading and spelling ability, pragmatic language and communication) and examining the pattern of associations with neuropsychiatric PRS (focussing on disorders where previous research suggests genetic overlap in childhood: ADHD, ASD, schizophrenia, depression and anxiety). We also explored whether the patterns of association were different when using self‐reports as compared to parent‐reports.

## Method

### Sample

We analysed data from the Avon Longitudinal Study of Parents and Children (ALSPAC), a well‐established prospective, longitudinal birth cohort study. Details of this study are provided in Appendix S1.

### ADHD and ASD symptoms

At age 25 years, young adult ADHD symptoms were measured using the parent‐rated 5‐item ADHD subscale of the Strengths and Difficulties Questionnaire (SDQ) (Goodman, 1997) (range: 0–10) and ASD symptoms using the parent‐rated 12‐item Social Communication Disorders Checklist (SCDC) (Skuse, Mandy, & Scourfield, 2005) (range: 0–24). Childhood ADHD and ASD symptoms were assessed using the same parent‐rated measures at age 11/12 years. Self‐rated symptom reports from the adults themselves were additionally obtained at age 25 years using the same 5‐item subscale of the SDQ (Goodman, 1997) for ADHD and the 50‐item Autism Spectrum Quotient (AQ) (Baron‐Cohen, Wheelwright, Skinner, Martin, & Clubley, 2001) (range: 0–50) for ASD symptoms.

Our primary measures are continuous, although we also report the proportion of individuals with 'high' symptoms for descriptive purposes. Elevated ADHD symptoms were defined using the childhood/adolescence recommended cut points of ≥8 for the parent‐rated SDQ and ≥7 for the self‐rated SDQ (Goodman, 1997) as there is currently no research to guide appropriate cut points for adult ADHD. We used the cut point of ≥9 for the parent‐rated SCDC (Skuse et al., 2005) and the recommended ≥32 cut point for the AQ (Baron‐Cohen et al., 2001).

### Other neurodevelopmental problems

Based on the DSM‐5 conceptualisation of neurodevelopmental disorders (American Psychiatric Association, 2013), we investigated the following neurodevelopmental problems in addition to ADHD and ASD: IQ, reading, spelling, pragmatic language and communication.

IQ was assessed using the Wechsler Intelligence Scale for Children (Wechsler, Golombok, & Rust, 1992) at age 8 years. Childhood reading ability was measured using the Wechsler Objective Reading Dimensions (Rust, Golombok, & Trickey, 1993) and spelling ability based on 15 age‐appropriate words developed by Nunes and Bryant (Nunes, Bryant, & Bindman, 1997) at age 7 years. Childhood pragmatic language was assessed using the parent‐reported Children's Communication Checklist (CCC) (Bishop, 1998) at age 9 years, which is derived as the sum of 5 subscales: inappropriate initiation, coherence, stereotyped conversation, use of conversational context and conversational rapport. Young adult communication was assessed using the parent‐rated Communication Checklist – Adult (CC‐A) (Whitehouse, Coon, Miller, Salisbury, & Bishop, 2010) at age 25 years, which includes the three subscales: language structure, pragmatic skills and social engagement. The CC‐A was reverse‐coded so that higher scores reflect better communication skills.

### Polygenic risk scores

Polygenic risk scores (PRS) were generated using PRSice version 1.25 (Euesden, Lewis, & O'Reilly, 2015) based on GWAS of ADHD (Demontis et al., 2019), ASD (Grove et al., 2019), schizophrenia (Pardinas et al., 2018), depression (Wray et al., 2018) and anxiety (Purves et al., 2019). Genotyping details as well as full methods for generating the PRS are presented in Appendix S2.

### Analyses

Individuals with parent‐rated or self‐rated ADHD and/or ASD data in young adulthood were included in our analyses (*N* = 6,064). Missing data were addressed by combining imputation with inverse probability weighting (Seaman, White, Copas, & Li, 2012), which is described in Appendix S3. Sensitivity analyses using alternative methods for addressing missing data including using multiple imputation for the full ALSPAC sample (i.e. including those with and without young adult data, *N* = 14,692), using inverse probability weighting without multiple imputation and using complete cases are also described in Appendix S3.

Associations between ADHD and ASD symptoms and with other neurodevelopmental problems were examined using separate univariable regressions with ADHD/ASD symptoms as the outcome. Multivariable regression was used to investigate associations with PRS for ADHD, ASD, schizophrenia, depression and anxiety diagnoses simultaneously (i.e. to focus on variance explained by the specific PRS rather than that shared across the PRS); sensitivity analyses were conducted to investigate associations for each of the five PRS separately. Both unstandardised coefficients (i.e. the ADHD/ASD score increase per one unit change in the exposure variable) and standardised (beta) coefficients (i.e. the ADHD/ASD standard deviation increase per standard deviation change in the exposure variable: generated by standardising variables to mean = 0 and standard deviation = 1) are presented. Sensitivity analyses were conducted stratifying analyses by sex and for persistent/late‐onset ADHD. Late‐onset ADHD was defined using our previous published criterion of symptom onset after age 12 years, based on 4 childhood assessments using the recommended cut points (see Appendix S4) (Cooper et al., 2018).

## Results

ADHD and ASD mean scores at age 25 years and the proportion of individuals with high symptoms (for descriptive purposes only) are shown in Table 1. Both parent‐rated and self‐rated ADHD symptoms using the SDQ were higher in males than females (mean difference = 0.37, 95% CI = 0.24–0.50, *p* = 4 × 10^−08^ and mean difference = 0.49, 95% CI = 0.34–0.64, *p* = 3 × 10^−10^, respectively). Parent‐rated (SCDC) and self‐rated (AQ) ASD scores were also associated with male sex (mean SCDC sex difference = 0.26, 95% CI = −0.01–0.50, *p* = .04 and mean AQ difference = 2.01, 95% CI = 1.53–2.49, *p* = 5 × 10^−16^, respectively).

**Table 1:** Descriptive statistics: ADHD and ASD symptoms at age 25 years (*N* = 6,064)

	Mean (95% CI)			High symptoms (95% CI)		
	Whole sample	Males	Females	Whole sample	Males	Females
ADHD symptoms						
Parent‐rated (SDQ)	1.76 (1.70–1.82)	1.95 (1.84–2.05)	1.58 (1.50–1.66)	1.2% (0.8–1.6)	1.9% (1.2–2.6)	0.5% (0.2–0.8)
Self ‐rated (SDQ)	3.31 (3.23–3.39)	3.55 (3.43–3.68)	3.06 (2.98–3.15)	9.4% (8.3–10.5)	11.9% (10.0–13.8)	6.8% (5.8–7.9)
ASD symptoms						
Parent‐rated (SCDC)	1.89 (1.77–2.01)	2.02 (1.89–2.21)	1.76 (1.61–1.92)	5.6% (4.7–6.4)	6.7% (5.3–8.1)	4.4% (3.4–5.3)
Self ‐rated (AQ)	17.36 (17.11–17.61)	18.36 (17.96–18.76)	16.35 (16.06–16.64)	3.1% (2.5–3.8)	3.8% (2.7–5.0)	2.4% (1.8–3.1)

### Association with neurodevelopmental problems

Parent‐rated ADHD and ASD symptoms in young adults were strongly correlated (*r* = 0.61, 95% CI = 0.57–0.64, *p* = 1 × 10^−211^). Parent‐rated ADHD symptoms in adulthood were moderately correlated with parent‐reported ADHD and ASD symptoms at age 11/12 years (childhood ADHD *r* = 0.51, 95% CI = 0.47–0.54, *p* = 8 × 10^−141^ and childhood ASD *r* = 0.39, 95% CI = 0.35–0.43, *p* = 1 × 10^−77^), as were adult parent‐rated ASD symptoms (childhood ADHD *r* = 0.33, 95% CI = 0.29–0.38, *p* = 2 × 10^−48^ and childhood ASD *r* = 0.43, 95% CI = 0.38–0.48, *p* = 2 × 10^−66^).

Associations between adult ADHD and ASD symptoms and other neurodevelopmental problems are shown in Table 2; associations for childhood ADHD and ASD symptoms in ALSPAC are shown in Table S1 for comparison. At age 25 years, there was strong evidence of both parent‐rated ADHD and ASD symptoms being associated with lower childhood IQ, reading and spelling ability, pragmatic language and communication problems in adult life.

**Table 2:** Associations between other neurodevelopmental problems and young adult ADHD/ASD symptoms

	ADHD symptoms				ASD symptoms			
	*B*	β	(95% CI)	*p*	*B*	β	(95% CI)	*p*
Parent‐reported symptoms in young adulthood								
IQ: age 8	−.03	−.24	(−0.28, −0.20)	3 × 10^−30^	−.04	−.20	(−0.25, −0.16)	2 × 10^−19^
Reading: age 7	−.18	−.22	(−0.26, −0.18)	3 × 10^−26^	−.27	−.19	(−0.23, −0.14)	2 × 10^−16^
Spelling: age 7	−.13	−.24	(−0.27, −0.20)	2 × 10^−30^	−.19	−.19	(−0.23, −0.15)	3 × 10^−17^
Pragmatic language: age 9	−.09	−.38	(−0.42, −0.34)	1 × 10^−64^	−.17	−.38	(−0.43, −0.34)	2 × 10^−57^
Communication: age 25	−.05	−.48	(−0.54, −0.42)	3 × 10^−53^	−.13	−.60	(−0.66, −0.55)	1 × 10^−104^
Self‐reported symptoms in young adulthood								
IQ: age 8	−.01	−.08	(−0.12, −0.04)	2 × 10^−04^	−.06	−.14	(−0.18, −0.10)	5 × 10^−12^
Reading: age 7	−.09	−.09	(−0.13, −0.05)	2 × 10^−05^	−.37	−.12	(−0.16, −0.08)	1 × 10^−09^
Spelling: age 7	−.07	−.11	(−0.16, −0.07)	8 × 10^−08^	−.26	−.12	(−0.16, −0.08)	7 × 10^−10^
Pragmatic language: age 9	−.05	−.17	(−0.22, −0.13)	6 × 10^−15^	−.21	−.23	(−0.27, −0.19)	5 × 10^−27^
Communication: age 25	−.02	−.17	(−0.22, −0.12)	8 × 10^−12^	−.12	−.28	(−0.32, −0.23)	5 × 10^−30^

Self‐rated ADHD and ASD symptoms at age 25 years were also correlated with each other (*r* = 0.36, 95% CI = 0.33–0.40, *p* = 2 × 10^−79^) and with other neurodevelopmental problems (also shown in Table 2): the magnitudes of the associations were generally smaller than observed for parent‐rated symptoms, although for ASD symptoms some of the confidence intervals for the different informants were overlapping.

### Association with neuropsychiatric genetic risk scores

Associations with PRS are shown in Table 3. Both parent‐rated ADHD and ASD symptoms in adulthood were associated with ADHD PRS; associations with childhood ADHD and ASD symptoms are shown in Table S1 for comparison. Self‐reported ADHD symptoms were also associated with ADHD PRS but showed additional association with depression PRS. We did not find strong evidence of association with any neuropsychiatric PRS for self‐rated ASD symptoms: neither parent‐ nor self‐rated ASD symptoms were associated with higher ASD PRS. Sensitivity analyses examining the five PRS separately (univariable analyses) showed a similar pattern of results (Table S2), with the exception that the association with depression and schizophrenia PRS was stronger for parent‐rated ASD symptoms when the other PRS were not included in the model.

**Table 3:** Associations between neuropsychiatric genetic risk scores and parent‐rated ADHD/ASD symptoms in young adulthood

	ADHD symptoms				ASD symptoms			
	*B*	β	(95% CI)	*p*	*B*	β	(95% CI)	*p*
Parent‐reported symptoms in young adulthood								
ADHD PRS	.23	.12	(0.08, 0.16)	7 × 10^−08^	.33	.10	(0.05, 0.14)	2 × 10^−05^
ASD PRS	−.06	−.03	(−0.08, 0.01)	.14	−.13	−.04	(−0.08, 0.00)	.07
Schizophrenia PRS	.09	.05	(0.01, 0.09)	.02	.21	.06	(0.02, 0.11)	.01
Depression PRS	.02	.01	(−0.03, 0.06)	.56	.16	.05	(0.00, 0.09)	.04
Anxiety PRS	.01	.01	(−0.04, 0.05)	.78	.06	.02	(−0.03, 0.06)	.45
Self‐reported symptoms in young adulthood								
ADHD PRS	.16	.08	(0.03, 0.12)	5 × 10^−04^	.19	.03	(−0.01, 0.07)	.20
ASD PRS	−.11	−.05	(−0.10, −0.01)	.01	−.02	−.00	(−0.05, 0.04)	.92
Schizophrenia PRS	.04	.02	(−0.02, 0.06)	.41	.29	.04	(0.00, 0.08)	.05
Depression PRS	.14	.07	(0.02, 0.11)	3 × 10^−03^	.32	.05	(0.00, 0.09)	.04
Anxiety PRS	.10	.05	(0.01, 0.09)	.02	.24	.04	(−0.01, 0.08)	.10

PRS, polygenic risk score. Across imputation data sets: parent‐rated ADHD mean *R* ^2^ = .017 (range = 0.009–0.029); parent‐rated ASD mean *R* ^2^ = .017 (range = 0.008–0.029); self‐rated ADHD mean *R* ^2^ = .016 (range = 0.009–0.027); self‐rated ASD mean *R* ^2^ = .008 (range = 0.001–0.017).

### Sensitivity analyses: stratifying by sex and persistent/late‐onset ADHD

Sensitivity analyses did not suggest that associations observed for ADHD and ASD in our primary analyses differed in males and females (Tables S3 and S4).

Investigating associations separately for persistent and late‐onset ADHD found somewhat stronger associations with male sex, other neurodevelopmental problems and ADHD PRS for persistent ADHD; in particular, self‐rated late‐onset ADHD was not associated with other neurodevelopmental problems (Tables S5 and S6), although confidence intervals were largely overlapping and given the small sample size for the categorical definitions, statistical power to detect association was low.

### Sensitivity analyses: missing data

Male sex, other neurodevelopmental problems and higher neuropsychiatric PRS were associated with missing data in adulthood (Table S7). Sensitivity analyses using different approaches to handle missing data found a similar pattern of associations (Tables S8–S11).

## Discussion

This study aimed to examine ADHD and ASD traits in young adulthood in a UK population birth cohort. Specifically, we explored associations with other neurodevelopmental problems and genetic risk for neuropsychiatric disorders using many of the same measures and informants as were used in childhood.

For adults, parent‐reported ADHD traits behaved in a similar fashion to those reported in childhood; we observed strong associations with current ASD symptoms and communication problems as well as with lower childhood IQ, reading and spelling ability and pragmatic language. As far as we are aware, there have been no studies of adult ADHD symptoms that have investigated overlap with additional childhood neurodevelopmental problems. We also found ADHD traits to be associated with ADHD polygenic risk scores (PRS), without strong associations with the other neuropsychiatric PRS. This is consistent with previous childhood research (although this did not investigate anxiety PRS) (Demontis et al., 2019; Martin, Hamshere, Stergiakouli, O'Donovan, & Thapar, 2015; Riglin et al., 2019) and thus indicates that parents rate a similar ADHD construct in their offspring both in childhood and adult life. This suggests that, where available, parents – whose reports are typically no longer used after the age of 18 years – may still provide useful additional information in assessment clinics when first diagnosing adult ADHD.

Consistent with previous work (Biederman et al., 1994; Simon et al., 2009), we observed a weaker association with male sex for parent‐rated ADHD symptoms at age 25 years compared with that in childhood. Sensitivity analyses suggested this difference may be accounted for by the late‐onset ADHD group as persistent ADHD did show a male excess, while late onset did not. In keeping with previously reported findings in ALSPAC at age 17 years, late‐onset symptoms at age 25 years did not show the typical childhood neurodevelopmental profile observed for childhood‐onset persistent symptoms (Cooper et al., 2018) but interestingly were associated with poorer parent‐reported communication/language at age 25 years.

Self‐rated ADHD symptoms also were associated with earlier childhood neurodevelopmental problems, although less obviously so than parent‐reported ADHD. Also, the association between self‐rated ADHD and male sex was less marked in adult life than what is typically reported in childhood. However, self‐rated ADHD symptoms were similar to parent‐reported ADHD symptoms in that they showed association with ADHD PRS. Unlike parent‐rated ADHD symptoms, they also showed association with depression PRS. This raises the possibility that self‐endorsement of ADHD symptoms may be associated with additional genetic liabilities or that self‐ratings are less specific than parent ratings, although this would need further investigation. Sensitivity analyses suggest that this could be driven by (self‐rated) late‐onset ADHD, although differing associations with PRS for different raters could also be driven in part by rater effects in the discovery GWAS patient samples used to derive the PRS. For example, the ADHD and ASD PRS are derived from primarily childhood samples which will rely more heavily on parent ratings, compared with the schizophrenia, depression and anxiety PRS, which are derived from adult samples and therefore are based on interviews, clinical notes and self‐reports, all of which will predominantly reflect patient information rather than parents.

When defining categorical outcome measures, we noted that the rate of self‐reported adult ADHD was much higher than when using parent reports, and also higher than reported prevalence rates in epidemiological studies. The mean level of self‐reported (continuous) ADHD symptoms was also higher than parent‐rated symptoms, although the difference was less pronounced than for categorical definitions because the (childhood‐based) symptom cut point for a categorical outcome is lower for self‐reports than parent reports (Goodman, 1997). It is important to note that we defined categorical ADHD using the recommended parent‐ and self‐reported cut points for childhood/adolescence (lower for self‐reports), because there is currently no recommended cut point for adulthood. Had we used the same cut point for self‐reports as parent reports (≥8), this would have captured 4.3% of the sample which although still higher than the 1.2% observed when using parent reports, is a less pronounced difference (than 9.4% using the self‐report threshold of ≥7). While some research suggests that adults with ADHD are likely to under‐report their symptoms and impairment compared with parent reports (Barkley, Fischer, Smallish, & Fletcher, 2002), our findings suggest that for general population samples, self‐reported questionnaire‐based measures of ADHD may require a more stringent cut point in adult life for defining elevated symptoms, although this needs further investigation. As our analyses focussed on continuous measures of ADHD and ASD, the cut point used for categorical definitions of ADHD will not have affected our reported associations for ADHD/ASD traits; determining the most appropriate cut points in adulthood is nevertheless an important area for future research.

For ASD, both parent‐ and self‐rated adult ASD symptoms were associated with male sex, lower childhood IQ, reading and spelling scores and language and communication problems. While the magnitudes of the associations were somewhat smaller for self‐ than parent‐rated, this informant difference was less pronounced than was observed for ADHD symptoms – particularly for objective measures (IQ, reading, spelling). This is despite having different measures for each informant, because our primary aim was to utilise parent reports using the same measures used in childhood and a self‐reported version of the SCDC was not available: we used the self‐rated AQ. Previous work in ASD samples has called into question the validity of the self‐rated AQ, suggesting that for some individuals, self‐report responses may relate to factors other than ASD, such as lack of interest in social interactions (Bishop & Seltzer, 2012). While our research does not exclude this possibility, associations between self‐rated ASD symptoms and other neurodevelopmental problems suggest that in general population samples, self‐rated ASD measures can to some extent tap neurodevelopmental impairment.

Genetic findings for continuous measures of ASD have been mixed in previous studies, although as far as we know PRS associations with adult ASD traits have not been previously examined. As previously observed in childhood, parent‐rated but not self‐rated adult ASD symptoms were associated with ADHD PRS. Again, stronger associations for parent‐rated symptoms with ADHD PRS could be partly driven by a greater reliance on parent‐rated compared with self‐rated symptoms in the ADHD GWAS used to derive these PRS. Weak associations were additionally found for both parent‐ and self‐rated ASD symptoms with schizophrenia and depression PRS, with less strong evidence for anxiety PRS. Previous work in ALSPAC in childhood has shown association between ASD traits and schizophrenia PRS, particularly in adolescence (St Pourcain et al., 2018): our work suggests that strong associations may not extend into young adulthood. Sensitivity analyses with the PRS separately (univariable instead of multivariable analyses) uncovered stronger evidence of association with depression and schizophrenia PRS for parent‐rated ASD symptoms. This suggests that these associations are not independent of ADHD PRS (which is associated with parent‐rated ASD). Neither parent‐ or self‐rated ASD symptoms at age 25 years were associated with ASD PRS, nor did sensitivity analyses on age 12 ASD symptoms find association with ASD PRS. Previous work on children in ALSPAC reported association with ASD PRS for this same measure of ASD at age 8 years, but not at older ages (spanning 11–17 years), again suggesting that the genetic architecture of these traits may vary across development (St Pourcain et al., 2018). Another potential explanation for the lack of association with ASD PRS is that the ASD measure could be capturing phenotypic characteristics that are not necessarily autism‐like in an adult population cohort. Our null finding could also be due to the relatively small ASD discovery GWAS or the characteristics of cases and controls included in these studies: for example, unlike other neuropsychiatric PRS, ASD PRS are associated with higher cognitive ability (Grove et al., 2019). We did not investigate whether possible 'late‐onset' ASD may have affected findings, as such cases have not been highlighted (as they have for ADHD; Asherson & Agnew‐Blais, 2019): it was beyond the scope of this paper to investigate age at onset in detail, although this is a possible area for future research.

Overall, our findings add to discussion about the characteristics of ADHD and ASD in adulthood: we found that these traits mainly behave similarly in childhood and adulthood even when using scales validated in younger populations and with the switch to self‐report in adult life. However, self‐reported ADHD in general appeared to be less strongly associated with other neurodevelopmental problems, although late‐onset ADHD may have contributed to this observation. Our findings highlight the benefits of prospective studies that use the same measures and same rater overtime and across ages: had we only investigated self‐rated symptoms in adulthood, weaker associations with other neurodevelopmental problems could have been attributed to age rather than reporter. This suggests there may be potential benefits to gathering additional information from parents, where available, about their young adult offspring for clinical purposes, which merits further investigation.

Our findings should be considered in the light of several limitations. In particular, parents included in our sample had typically already completed multiple questionnaires about their offspring’s ADHD and ASD symptoms across childhood and adolescence (a median seven and four times for the SDQ and SCDC, respectively), which may have affected their ratings both through increased awareness of these traits and if current ratings were influenced by previous ratings. We were also reliant on repeated measures of ADHD and ASD symptoms that have yet to be validated in adulthood, including what the optimum cut points for defining high symptoms are. Also, the constellation of symptoms for ADHD and ASD may change with age. With regard to the genetic liability associations, PRS currently only explain a small proportion of genetic liability to psychiatric disorders (Schizophrenia Working Group of the Psychiatric Genomics Consortium, 2014). Our sensitivity analyses investigating late‐onset ADHD were underpowered and limited by uncertainty in classification for some individuals (e.g. those with subthreshold childhood symptoms): a comprehensive investigation of late‐onset symptoms was beyond the scope of this paper and the possible differences between late‐onset and persistent adult ADHD symptoms that we observed require further study. Like many longitudinal samples, ALSPAC also suffers from nonrandom attrition, whereby individuals at elevated risk of psychopathology and with higher neuropsychiatric PRS are more likely to drop out of the study (Martin et al., 2016; Taylor et al., 2018). We used multiple imputation with inverse probability weighting to try to minimise the effect of missingness, although this assumes that missingness is independent of the unobserved missing data (given the variables in the imputation model) and that the imputation model is correctly specified. Finally, our study examined young adulthood population ADHD and ASD traits: while there is evidence that ADHD and ASD symptoms behave as continuously distributed traits without clear‐cut thresholds in terms of associations with adverse outcome (Mandy & Lai, 2016; Robinson et al., 2016; Rutter et al., 2011; Thapar & Cooper, 2016), this research has focused on childhood. It is therefore not clear whether our findings about ADHD and ASD traits in young adulthood are generalisable to clinical diagnoses.

## Conclusion

ADHD and ASD symptoms in young adulthood generally show a similar pattern of association with other neurodevelopmental problems and genetic liability to ADHD as observed in childhood; associations were somewhat weaker for self‐reported adult ADHD and ASD symptoms. These findings support the validity of ADHD and ASD traits in adulthood including the use of self‐report, but also suggest that where available, parent reports even at age 25 may still be useful.

## Supporting information

**Table S1.** Associations between childhood ADHD/ASD at age 11/12 years and neurodevelopmental problems, neuropsychiatric genetic risk scores (PRS) and sex.
**Table S2.** Univariable associations between neuropsychiatric genetic risk scores and parent‐rated ADHD/ASD symptoms in young adulthood.
**Table S3.** Sensitivity analyses stratifying by sex for parent‐rated ADHD‐ASD symptoms in young adulthood: associations with neurodevelopmental problems and neuropsychiatric genetic risk scores.
**Table S4.** Sensitivity analyses stratifying by sex for self‐rated ADHD‐ASD symptoms in young‐adulthood: associations with neurodevelopmental problems and neuropsychiatric genetic risk scores.
**Table S5.** Sensitivity analyses investigating parent‐rated persistent and late‐onset ADHD symptoms in young‐adulthood: associations with sex, neurodevelopmental problems and neuropsychiatric genetic risk scores.
**Table S6.** Sensitivity analyses investigating self‐rated persistent and late‐onset ADHD symptoms in young‐adulthood: associations with sex, neurodevelopmental problems and neuropsychiatric genetic risk scores.
**Table S7.** Inspecting missingness: Associations between analysis variables and missing young‐adult ADHD/ASD data.
**Table S8.** Associations with parent‐rated ADHD symptoms in young adulthood using different approached to missing data.
**Table S9.** Associations with parent‐rated ASD symptoms in young adulthood using different approached to missing data.
**Table S10.** Associations with self‐rated ADHD symptoms in young adulthood using different approached to missing data.
**Table S11.** Associations with self‐rated ASD symptoms in young adulthood using different approached to missing data.
**Table S12.** Associations between variables include in the inverse probability weight and missing young‐adult ADHD/ASD data.
**Table S13.** Additional measures of ADHD/ASD included in the multiple imputation model: correlations with young‐adult ADHD/ASD measures.
