# Supplementary material for: Investigating attention‐deficit hyperactivity disorder and autism spectrum disorder traits in the general population: What happens in adult life?
Source: J Child Psychol Psychiatry. 2020 Jul 14;62(4):449–57. doi: 10.1111/jcpp.13297 (PMC8365432; doi:10.1111/jcpp.13297)
Supplement: Supplementary file 1 — Table S1. Associations between childhood ADHD/ASD at age 11/12 years and neurodevelopmental problems, neuropsychiatric genetic risk scores (PRS) and sex. Table S2. Univariable associations between neuropsychiatric genetic risk scores and parent‐rated ADHD/ASD symptoms in young adulthood. Table S3. Sensitivity analyses stratifying by sex for parent‐rated ADHD‐ASD symptoms in young adulthood: associations with neurodevelopmental problems and neuropsychiatric genetic risk scores. Table S4. Sensitivity analyses stratifying by sex for self‐rated ADHD‐ASD symptoms in young‐adulthood: associations with neurodevelopmental problems and neuropsychiatric genetic risk scores. Table S5. Sensitivity analyses investigating parent‐rated persistent and late‐onset ADHD symptoms in young‐adulthood: associations with sex, neurodevelopmental problems and neuropsychiatric genetic risk scores. Table S6. Sensitivity analyses investigating self‐rated persistent and late‐onset ADHD symptoms in young‐adulthood: associations with sex, neurodevelopmental problems and neuropsychiatric genetic risk scores. Table S7. Inspecting missingness: Associations between analysis variables and missing young‐adult ADHD/ASD data. Table S8. Associations with parent‐rated ADHD symptoms in young adulthood using different approached to missing data. Table S9. Associations with parent‐rated ASD symptoms in young adulthood using different approached to missing data. Table S10. Associations with self‐rated ADHD symptoms in young adulthood using different approached to missing data. Table S11. Associations with self‐rated ASD symptoms in young adulthood using different approached to missing data. Table S12. Associations between variables include in the inverse probability weight and missing young‐adult ADHD/ASD data. Table S13. Additional measures of ADHD/ASD included in the multiple imputation model: correlations with young‐adult ADHD/ASD measures. [file JCPP-62-449-s001.docx]

**Supplementary Material**

**The Avon Longitudinal Study of Parents and Children (ALSPAC)**

Pregnant women resident in Avon, UK with expected dates of delivery 1st April 1991 to 31st December 1992 were invited to take part in the study. The initial number of pregnancies enrolled is 14,541 (for these at least one questionnaire has been returned or a “Children in Focus” clinic had been attended by 19/07/99). Of these initial pregnancies, there was a total of 14,676 foetuses, resulting in 14,062 live births and 13,988 children who were alive at 1 year of age. When the oldest children were approximately 7 years of age, an attempt was made to bolster the initial sample with eligible cases who had failed to join the study originally. As a result, the total sample size for data collected after the age of seven is therefore 15,454 pregnancies, resulting in 15,589 foetuses. Of these 14,901 were alive at 1 year of age. Part of this data was collected using REDCap (https://projectredcap.org/resources/citations/). Ethical approval for the study was obtained from the ALSPAC Law and Ethics Committee and Local Research Ethics Committees. Informed consent for the use of data collected via questionnaires and clinics was obtained from participants following the recommendations of the ALSPAC Ethics and Law Committee at the time. Consent for biological samples has been collected in accordance with the Human Tissue Act (2004). Please note that the study website contains details of all the data that is available through a fully searchable data dictionary and variable search tool: http://www.bristol.ac.uk/alspac/researchers/our-data/. Further details of the study, measures and sample can be found elsewhere (Boyd et al., 2013; Fraser et al., 2013; Northstone et al., 2019). Where families included multiple births, we included the oldest sibling.

**Generating polygenic risk scores**

Polygenic risk scores (PRS) were derived for 9,912 ALSPAC children who were genotyped using the Illumina HumanHap500-quad genotyping array. Individuals were excluded on the basis of gender mismatches, minimal or excessive heterozygosity, genotype missingness (>3%), insufficient sample replication (IBD <0.8), non-European ancestry (assessed by multidimensional scaling analysis and compared with Hapmap II) and cryptic relatedness (IBD > 0.1). SNPs were excluded based on minor allele frequency (<1%), call rate (<95%) or evidence for violations of Hardy-Weinberg equilibrium (P < 5E-7). Imputation was conducted by the ALSPAC team using Impute V2.2.2 against the 1000 genomes reference panel (Phase 1, Version 3: all polymorphic SNPs excluding singletons), using all 2,186 reference haplotypes (including non-Europeans). Best guess SNPs were subsequently filtered based on minor allele frequency (<1%) and imputation quality (INFO<0.8).

Genome-wide association study (GWAS) were filtered to remove SNPs that were palindromic, insertions/deletions, non-autosomal, INFO score <0.8, missing in N>1 study and duplicates (https://github.com/ricanney). Depression results for 23andme (75,607 cases and 231,747 controls) (Hyde et al., 2016) and the other samples included in the latest depression GWAS (Wray et al., 2018) (PGC29, deCODE, Generation Scotland, GERA, iPSYCH, and UK Biobank) were meta-analysed in METAL.

PRS were generated for individuals in ALSPAC as the number of disorder risk alleles – defined using the GWAS summary statistics - weighted by effect size, using PRSice version 1.25 (Euesden, Lewis, & O'Reilly, 2015); SNPs were clumped with an R^2^ threshold of 0.1 and a distance threshold of 1000kb and excluding the extended major histocompatibility complex (MHC; chromosome 6: 26-33Mb) due to the high linkage disequilibrium (LD) within this region.

In the primary analyses we defined risk alleles as those associated with case-status at p<0.05 as this threshold has previously been shown to maximally capture phenotypic variance for schizophrenia (Schizophrenia Working Group of the Psychiatric Genomics Consortium, 2014). PRS were generated using GWAS of ADHD (19,099 cases and 34,194 controls) (Demontis et al., 2019), ASD (18,381 cases and 27,969 controls) (Grove et al., 2019), schizophrenia (40,675 cases and 64,643 controls) (Pardinas et al., 2018), depression (135,458 cases and 344,901 controls)(Wray et al., 2018) and anxiety (25,453 cases and 58,113 controls) (Purves et al., 2019).Polygenic risk scores were standardized using Z-score transformation.

**Missing data**

Missing data were addressed by combining imputation with inverse probability weighting (Seaman, White, Copas, & Li, 2012). Data were imputed for the N=6064 individuals in our sample, using inverse-probability weighting to address selection bias in this sample compared to the “full” ALSPAC sample (i.e. including those without parent- or self-rated ADHD and/or ASD data in young-adulthood: N=14692).

First, inverse probability weighting (IPW) was used to generate weights derived from a logistic regression analysis of missing young-adult ADHD/ASD data for a set of measures assessed in or soon after pregnancy with minimal missingness that were that showed independent association with missing data (see Supplementary Table 12). The Hosmer-Lemeshow test was used assess the fit of the missingness model; results did not indicate poor fit (Hosmer-Lemeshow χ2(10)=10.49, p=0.23). For the included sample (N=6064) weights ranged from 1.17 to 15.52.

Second, multiple imputation by chained equations (White, Royston, & Wood, 2011a) was used to impute missing data whereby variables that predicted young-adult ADHD/ASD data missingness (Supplementary Table 12), variables in the analysis models (Supplementary Table 9) (including the IPW weight) and measures of ADHD/ASD symptoms at different time-points (Supplementary Table 13), were included in an imputation model to generate 250 imputed datasets. This was estimated to be a sufficient number of imputations to ensure that standard errors would not change considerably if the data were imputed again (the recommended 2-stage quadratic rule based on the initial imputation of 250 datasets suggested 54-74 imputations were needed for PRS associations with adult symptoms, for which there were the most missing data) (von Hippel, 2018). For the primary analyses, estimates were combined across imputed datasets using Rubin’s rules (White, Royston, & Wood, 2011b) and a robust estimator was used due to uncertainty in weights (Seaman et al., 2012). Sensitivity analyses investigating the consistency of results using multiple imputation for the full ALSPAC sample, using IPW without multiple imputation and using complete cases found a similar pattern of associations (Supplementary Tables 8-11).

**Persistent and late-onset ADHD symptoms**

For sensitivity analyses, we defined persistent ADHD symptoms as those with high ADHD symptoms who were also estimated to have high symptoms in childhood (at age 12 years): estimated prevalence based on parent-reports was 0.5%. In-line with previous work on this same cohort (Cooper et al., 2018), we defined late-onset ADHD symptoms as those with high young-adult symptoms who were estimated to have “close to average” (<6) SDQ scores at ages 7, 8, 9 and 12 years old. In the current sample, 0.2% were estimated to have late-onset symptoms (individuals with subthreshold symptoms at age 12 years or with subthreshold/high symptoms at an earlier point in childhood were not categorised as either persistent or late-onset).

Finally, using the same approach but for self-rated ADHD symptoms in young-adulthood (parent-ratings in childhood), 0.1% of the sample were estimated to have persistent symptoms and 0.5% to have late-onset symptoms. Associations with male sex, other neurodevelopmental problems and genetic risk scores for high ADHD symptoms, persistent symptoms and late-onset symptoms are shown in Supplementary Tables 5 and 6 for parent-rated and self-rated symptoms respectively.

| **Supplementary Table 1.** Associations between childhood ADHD/ASD at age 11/12 years and neurodevelopmental problems, neuropsychiatric genetic risk scores (PRS) and sex | | | | | | | | | | |  |
| --- | --- | --- | --- | --- | --- | --- | --- | --- | --- | --- | --- |
|  | ADHD symptoms | | | |  | ASD symptoms | | | | |  |
|  | B | β | (95% CI) | p | |  | B | β | (95% CI) | p | |
| *Other neurodevelopmental problems* | | | | | | | | | | |  |
| IQ: age 8 | -0.03 | -0.24 | (-0.28, -0.21) | 2x10^-38^ | |  | -0.03 | -0.16 | (-0.21, -0.12) | 2x10^-11^ | |
| Reading: age 7 | -0.24 | -0.26 | (-0.29, -0.22) | 2x10^-41^ | |  | -0.25 | -0.18 | (-0.22, -0.13) | 7x10^-13^ | |
| Spelling: age 7 | -0.20 | -0.30 | (-0.33, -0.26) | 3x10^-58^ | |  | -0.20 | -0.20 | (-0.25, -0.15) | 2x10^-15^ | |
| Pragmatic language: age 9 | -0.13 | -0.45 | (-0.49, -0.42) | 5x10^-125^ | |  | -0.24 | -0.53 | (-0.58, -0.48) | 2x10^-90^ | |
| Language: age 25 | -0.04 | -0.29 | (-0.32, -0.25) | 2x10^-48^ | |  | -0.08 | -0.39 | (-0.44, -0.33) | 8x10^-41^ | |
| *Genetic risk scores^*^* | | | | | | | | | | |  |
| ADHD PRS | 0.24 | 0.11 | (0.07, 0.15) | 8x10^-08^ | |  | 0.36 | 0.11 | (0.06, 0.16) | 3x10^-06^ | |
| ASD PRS | -0.09 | -0.04 | (-0.08, -0.00) | 0.04 | |  | -0.03 | -0.01 | (-0.06, 0.04) | 0.67 | |
| Schizophrenia PRS | 0.04 | 0.02 | (-0.02, 0.06) | 0.39 | |  | 0.11 | 0.03 | (-0.01, 0.08) | 0.17 | |
| Depression PRS | -0.00 | -0.00 | (-0.04, 0.04) | 0.94 | |  | -0.06 | -0.02 | (-0.07, 0.03) | 0.42 | |
| Anxiety PRS | 0.01 | 0.01 | (-0.03, 0.05) | 0.73 | |  | 0.06 | 0.02 | (-0.03, 0.06) | 0.42 | |
| *^*^*Multivariable analyses. PRS = polygenic risk score.  ***Sex differences***  ADHD: male mean=3.23, SE=0.06; female mean=2.38, SE=0.04; mean difference=0.85, 95% CI=0.71-0.99, p=1x10^-31^. ASD: male mean=2.73, SE=0.10; female mean=2.04, SE=0.7; mean difference=0.70, 95% CI=0.46-0.94, p=1x10^-08^. | | | | | | | | | | |  |

| **Supplementary Table 2.** Univariable associations between neuropsychiatric genetic risk scores and parent-rated ADHD/ASD symptoms in young adulthood | | | | | | | | | | | | |
| --- | --- | --- | --- | --- | --- | --- | --- | --- | --- | --- | --- | --- |
|  | ADHD symptoms | | | | |  |  | ASD symptoms | | | | |
|  | β | (95% CI) | p | Mean R^2^ | R^2^ range |  |  | β | (95% CI) | p | Mean R^2^ | R^2^ range |
| *Parent-reported symptoms in young adulthood* | | | | | | | | | | | | |
| ADHD PRS | 0.12 | (0.07, 0.16) | 9x10^-08^ | 0.013 | 0.005-0.023 |  |  | 0.10 | (0.05, 0.14) | 2x10^-05^ | 0.009 | 0.003-0.018 |
| ASD PRS | -0.00 | (-0.04, 0.04) | 0.91 | 0.000 | 0.000-0.002 |  |  | -0.01 | (-0.05, 0.03) | 0.62 | 0.000 | 0.000-0.002 |
| Schizophrenia PRS | 0.05 | (0.01, 0.10) | 0.01 | 0.003 | 0.000-0.008 |  |  | 0.07 | (0.03, 0.11) | 2x10^-03^ | 0.005 | 0.001-0.012 |
| Depression PRS | 0.03 | (-0.02, 0.07) | 0.21 | 0.001 | 0.000-0.004 |  |  | 0.06 | (0.02, 0.10) | 6x10^-03^ | 0.004 | 0.000-0.009 |
| Anxiety PRS | 0.01 | (-0.03, 0.06) | 0.60 | 0.000 | 0.000-0.004 |  |  | 0.03 | (-0.02, 0.07) | 0.23 | 0.001 | 0.000-0.005 |
| *Self-reported symptoms in young adulthood* | | | | | | | | | | | | |
| ADHD PRS | 0.07 | (0.03, 0.11) | 1x10^-03^ | 0.005 | 0.001-0.012 |  |  | 0.03 | (-0.01, 0.07) | 0.10 | 0.001 | 0.000-0.005 |
| ASD PRS | -0.03 | (-0.07, 0.01) | 0.16 | 0.001 | 0.000-0.005 |  |  | 0.01 | (-0.03, 0.05) | 0.63 | 0.000 | 0.000-0.003 |
| Schizophrenia PRS | 0.02 | (-0.02, 0.06) | 0.25 | 0.001 | 0.000-0.004 |  |  | 0.05 | (0.01, 0.09) | 0.03 | 0.002 | 0.000-0.018 |
| Depression PRS | 0.08 | (0.03, 0.12) | 4x10^-04^ | 0.006 | 0.001-0.012 |  |  | 0.06 | (0.01, 0.10) | 0.01 | 0.003 | 0.000-0.011 |
| Anxiety PRS | 0.06 | (0.02, 0.10) | 0.01 | 0.003 | 0.000-0.010 |  |  | 0.04 | (0.00, 0.09) | 0.04 | 0.002 | 0.000-0.008 |
| R^2^ values presented as the mean and range across the 250 imputed datasets. | | | | | | | | | | | | |

| **Supplementary Table 3.** Sensitivity analyses stratifying by sex for parent-rated ADHD-ASD symptoms in young-adulthood: associations with neurodevelopmental problems and neuropsychiatric genetic risk scores | | | | | | | | | | | | | | | | |
| --- | --- | --- | --- | --- | --- | --- | --- | --- | --- | --- | --- | --- | --- | --- | --- | --- |
|  | Males (N=2566) | | | | | | |  |  | Females (N=3498) | | | | | | |
|  | ADHD symptoms | | |  | ASD symptoms | | |  |  | ADHD symptoms | | |  | ASD symptoms | | |
|  | β | (95% CI) | p |  | β | (95% CI) | p |  |  | β | (95% CI) | p |  | β | (95% CI) | p |
| *Other neurodevelopmental problems* | | | | | | | | | | | | | | | | |
| IQ: age 8 | -0.25 | (-0.31, -0.19) | 3x10^-17^ |  | -0.20 | (-0.26, -0.13) | 2x10^-09^ |  |  | -0.23 | (-0.28, -0.18) | 2x10^-19^ |  | -0.20 | (-0.25, -0.14) | 5x10^-12^ |
| Reading: age 7 | -0.22 | (-0.28, -0.17) | 3x10^-14^ |  | -0.20 | (-0.26, -0.13) | 2x10^-09^ |  |  | -0.20 | (-0.25, -0.15) | 5x10^-14^ |  | -0.17 | (-0.23, -0.11) | 3x10^-09^ |
| Spelling: age 7 | -0.24 | (-0.30, -0.18) | 6x10^-16^ |  | -0.19 | (-0.26, -0.13) | 3x10^-09^ |  |  | -0.22 | (-0.27, -0.17) | 3x10^-17^ |  | -0.18 | (-0.23, -0.12) | 2x10^-10^ |
| Pragmatic language: age 9 | -0.40 | (-0.46, -0.33) | 1x10^-31^ |  | -0.42 | (-0.48, -0.35) | 3x10^-33^ |  |  | -0.35 | (-0.39, -0.30) | 1x10^-45^ |  | -0.34 | (-0.40, -0.28) | 3x10^-30^ |
| Language: age 25 | -0.48 | (-0.58, -0.39) | 8x10^-121^ |  | -0.63 | (-0.71, -0.54) | 3x10^-46^ |  |  | -0.47 | (-0.51, -0.42) | 2x10^-80^ |  | -0.57 | (-0.63, -0.52) | 3x10^-83^ |
| *Genetic risk scores^*^* |  |  |  |  |  |  |  |  |  |  |  |  |  |  |  |  |
| ADHD PRS | 0.12 | (0.06, 0.18) | 1x10^-04^ |  | 0.10 | (0.03, 0.16) | 3x10^-03^ |  |  | 0.12 | (0.07, 0.18) | 5x10^-06^ |  | 0.10 | (0.05, 0.16) | 4x10^-04^ |
| ASD PRS | -0.04 | (-0.10, 0.03) | 0.24 |  | -0.05 | (-0.11, 0.01) | 0.12 |  |  | -0.03 | (-0.08, 0.02) | 0.30 |  | -0.03 | (-0.08, 0.02) | 0.23 |
| Schizophrenia PRS | 0.04 | (-0.02, 0.10) | 0.17 |  | 0.08 | (0.01, 0.14) | 0.02 |  |  | 0.03 | (-0.02, 0.09) | 0.19 |  | 0.04 | (-0.02, 0.09) | 0.15 |
| Depression PRS | 0.02 | (-0.05, 0.08) | 0.67 |  | 0.04 | (-0.02, 0.10) | 0.022 |  |  | 0.02 | (-0.04, 0.07) | 0.57 |  | 0.06 | (0.00, 0.11) | 0.05 |
| Anxiety PRS | 0.03 | (-0.04, 0.09) | 0.42 |  | 0.03 | (-0.04, 0.09) | 0.42 |  |  | -0.02 | (-0.07, 0.03) | 0.49 |  | 0.01 | (-0.05, 0.06) | 0.82 |
| *^*^*Multivariable analyses | | | | | | | | | | | | | | | | |

| **Supplementary Table 4.** Sensitivity analyses stratifying by sex for self-rated ADHD-ASD symptoms in young-adulthood: associations with neurodevelopmental problems and neuropsychiatric genetic risk scores | | | | | | | | | | | | | | | | |
| --- | --- | --- | --- | --- | --- | --- | --- | --- | --- | --- | --- | --- | --- | --- | --- | --- |
|  | Males (N=2566) | | | | | | |  |  | Females (N=3498) | | | | | | |
|  | ADHD symptoms | | |  | ASD symptoms | | |  |  | ADHD symptoms | | |  | ASD symptoms | | |
|  | β | (95% CI) | p |  | β | (95% CI) | p |  |  | β | (95% CI) | p |  | β | (95% CI) | p |
| *Other neurodevelopmental problems* | | | | | | | | | | | | | | | | |
| IQ: age 8 | -0.08 | (-0.14, -0.02) | 0.01 |  | -0.13 | (-0.19, -0.07) | 4x10^-05^ |  |  | -0.09 | (-0.13, -0.04) | 3x10^-04^ |  | -0.17 | (-0.22, -0.13) | 4x10^-13^ |
| Reading: age 7 | -0.09 | (-0.15, -0.02) | 0.01 |  | -0.11 | (-0.16, -0.05) | 2x10^-04^ |  |  | -0.09 | (-0.13, -0.04) | 2x10^-04^ |  | -0.13 | (-0.17, -0.08) | 1x10^-07^ |
| Spelling: age 7 | -0.11 | (-0.17, -0.04) | 1x10^-03^ |  | -0.10 | (-0.16, -0.04) | 6x10^-05^ |  |  | -0.11 | (-0.15, -0.06) | 4x10^-06^ |  | -0.12 | (-0.17, -0.08) | 1x10^-07^ |
| Pragmatic language: age 9 | -0.18 | (-0.24, -0.11) | 1x10^-07^ |  | -0.22 | (-0.28, -0.16) | 8x10^-13^ |  |  | -0.15 | (-0.20, -0.11) | 2x10^-11^ |  | -0.21 | (-0.26, -0.17) | 2x10^-18^ |
| Language: age 25 | -0.17 | (-0.24, -0.10) | 5x10^-06^ |  | -0.27 | (-0.34, -0.20) | 3x10^-14^ |  |  | -0.16 | (-0.21, -0.11) | 1x10^-10^ |  | -0.28 | (-0.33, -0.23) | 1x10^-25^ |
| *Genetic risk scores^*^* |  |  |  |  |  |  |  |  |  |  |  |  |  |  |  |  |
| ADHD PRS | 0.09 | (0.03, 0.16) | 0.01 |  | 0.02 | (-0.04, 0.07) | 0.42 |  |  | 0.06 | (0.01, 0.11) | 0.01 |  | 0.03 | (-0.02, 0.08) | 0.18 |
| ASD PRS | -0.07 | (-0.12, 0.00) | 0.06 |  | -0.01 | (-0.07, 0.04) | 0.80 |  |  | -0.05 | (-0.10, 0.00) | 0.07 |  | 0.01 | (-0.05, 0.06) | 0.82 |
| Schizophrenia PRS | -0.01 | (-0.07, 0.05) | 0.79 |  | 0.02 | (-0.03, 0.08) | 0.41 |  |  | 0.01 | (-0.03, 0.06) | 0.56 |  | 0.02 | (-0.03, 0.07) | 0.37 |
| Depression PRS | 0.07 | (0.02, 0.14) | 0.02 |  | 0.05 | (-0.01, 0.09) | 0.09 |  |  | 0.06 | (0.01, 0.11) | 0.02 |  | 0.04 | (-0.01, 0.10) | 0.09 |
| Anxiety PRS | 0.06 | (0.01, 0.12) | 0.06 |  | 0.02 | (-0.04, 0.08) | 0.49 |  |  | 0.04 | (-0.01, 0.08) | 0.15 |  | 0.05 | (-0.00, 0.10) | 0.06 |
| *^*^*Multivariable analyses | | | | | | | | | | | | | | | | |

| **Supplementary Table 5.** Sensitivity analyses investigating parent-rated persistent and late-onset ADHD symptoms in young-adulthood: associations with sex, neurodevelopmental problems and neuropsychiatric genetic risk scores | | | | | | | | | | | | |
| --- | --- | --- | --- | --- | --- | --- | --- | --- | --- | --- | --- | --- |
|  |  | Adult ADHD (binary) estimated prevalence: 1.2% (N~73) | | |  | Persistent ADHD estimated prevalence: 0.5% (N~30) | | |  | Late-onset ADHD estimated prevalence: 0.2% (N~12) | | |
|  |  | OR | (95% CI) | p |  | OR | (95% CI) | p |  | OR | (95% CI) | p |
| Male sex |  | 4.15 | (2.05, 8.39) | 8x10^-05^ |  | 5.15 | (1.72, 15.46) | 3x10^-03^ |  | 1.75 | (0.37, 8.35) | 0.49 |
| *Other neurodevelopmental problems* | | | | | | | | | | | | |
| IQ: age 8 |  | 0.96 | (0.93, 0.98) | 6x10^-04^ |  | 0.96 | (0.93, 0.98) | 3x10^-03^ |  | 0.99 | (0.94, 1.04) | 0.72 |
| Reading: age 7 |  | 0.79 | (0.71, 0.90) | 5x10^-04^ |  | 0.75 | (0.64, 0.88) | 1x10^-03^ |  | 1.20 | (0.75, 1.93) | 0.45 |
| Spelling: age 7 |  | 0.83 | (0.76, 0.91) | 1x10^-04^ |  | 0.79 | (0.71, 0.89) | 1x10^-04^ |  | 1.08 | (0.84, 1.37) | 0.55 |
| Pragmatic language: age 9 |  | 0.90 | (0.88, 0.92) | 1x10^-18^ |  | 0.88 | (0.86, 0.91) | 3x10^-18^ |  | 1.00 | (0.90, 1.12) | 0.93 |
| Language: age 25 |  | 0.95 | (0.94, 0.97) | 2x10^-08^ |  | 0.96 | (0.94, 0.98) | 5x10^-06^ |  | 0.97 | (0.95, 0.98) | 1x10^-04^ |
| *Genetic risk scores^*^* |  |  |  |  |  |  |  |  |  |  |  |  |
| ADHD PRS |  | 1.32 | (0.90, 1.94) | 0.15 |  | 1.74 | (0.92, 3.27) | 0.09 |  | 0.97 | (0.41, 2.30) | 0.95 |
| ASD PRS |  | 0.80 | 0.57, 1.12) | 0.19 |  | 0.77 | (0.41, 1.41) | 0.39 |  | 0.76 | (0.35, 1.65) | 0.48 |
| Schizophrenia PRS |  | 1.28 | (0.91, 1.81) | 0.15 |  | 1.23 | (0.70, 2.17) | 0.47 |  | 1.24 | (0.61, 2.55) | 0.55 |
| Depression PRS |  | 0.98 | (0.69, 1.40) | 0.92 |  | 0.84 | (0.45, 1.57) | 0.59 |  | 1.19 | (0.60, 2.36) | 0.62 |
| Anxiety PRS |  | 1.25 | (0.87, 1.79) | 0.23 |  | 1.38 | (0.77, 2.46) | 0.28 |  | 1.52 | (0.60, 3.84) | 0.38 |
| *^*^*Multivariable analyses. Reference group = low adult symptoms. Approximate Ns based on estimated prevalence (combined across multiple imputed datasets). | | | | | | | | | | | | |

| **Supplementary Table 6.** Sensitivity analyses investigating self-rated persistent and late-onset ADHD symptoms in young-adulthood: associations with sex, neurodevelopmental problems and neuropsychiatric genetic risk scores | | | | | | | | | | | | |
| --- | --- | --- | --- | --- | --- | --- | --- | --- | --- | --- | --- | --- |
|  |  | Adult ADHD (binary)  estimated prevalence: 9.4% (N~570) | | |  | Persistent ADHD  estimated prevalence: 1.3% (N~79) | | |  | Late-onset ADHD  estimated prevalence: 4.7% (N~285) | | |
|  |  | OR | (95% CI) | p |  | OR | (95% CI) | p |  | OR | (95% CI) | p |
| Male sex |  | 1.84 | (1.44, 2.35) | 1x10^-06^ |  | 3.35 | (1.63, 6.88) | 1x10^-03^ |  | 1.18 | (0.85, 1.65) | 0.32 |
| *Other neurodevelopmental problems* | | | | | | | | | | | | |
| IQ: age 8 |  | 0.99 | (0.98, 1.00) | 0.01 |  | 0.96 | (0.94, 0.98) | 1x10^-03^ |  | 1.00 | (0.99, 1.01) | 0.53 |
| Reading: age 7 |  | 0.93 | (0.87, 0.98) | 0.01 |  | 0.76 | (0.67, 0.85) | 4x10^-06^ |  | 1.04 | (0.96, 1.12) | 0.36 |
| Spelling: age 7 |  | 0.93 | (0.89, 0.97) | 3x10^-04^ |  | 0.81 | (0.74, 0.89) | 4x10^-06^ |  | 1.00 | (0.95, 1.06) | 0.87 |
| Pragmatic language: age 9 |  | 0.96 | (0.94, 0.97) | 1x10^-07^ |  | 0.90 | (0.87, 0.92) | 8x10^-16^ |  | 1.02 | (1.00, 1.05) | 0.08 |
| Language: age 25 |  | 0.98 | (0.97, 0.99) | 2x10^-07^ |  | 0.96 | (0.95, 0.98) | 5x10^-07^ |  | 1.00 | (0.99, 1.00) | 0.32 |
| *Genetic risk scores^*^* |  |  |  |  |  |  |  |  |  |  |  |  |
| ADHD PRS |  | 1.22 | (1.05, 1.42) | 0.01 |  | 1.60 | (1.09, 2.33) | 0.02 |  | 1.09 | (0.90, 1.31) | 0.38 |
| ASD PRS |  | 0.89 | (0.77, 1.03) | 0.13 |  | 0.85 | (0.58, 1.26) | 0.43 |  | 0.89 | (0.75, 1.07) | 0.22 |
| Schizophrenia PRS |  | 1.04 | (0.91, 1.18) | 0.60 |  | 1.09 | (0.75, 1.58) | 0.66 |  | 1.01 | (0.86, 1.20) | 0.87 |
| Depression PRS |  | 1.19 | (1.03, 1.37) | 0.02 |  | 0.97 | (0.67, 1.42) | 0.89 |  | 1.18 | (0.98, 1.41) | 0.08 |
| Anxiety PRS |  | 1.09 | (0.95, 1.24) | 0.23 |  | 1.10 | (0.75, 1.62) | 0.61 |  | 1.12 | (0.94, 1.34) | 0.19 |
| *^*^*Multivariable analyses. Definitions based on parent-rated symptoms in childhood. Reference group = low adult symptoms. Approximate Ns based on estimated prevalence (combined across multiple imputed datasets). | | | | | | | | | | | | |

| **Supplementary Table 7.** Inspecting missingness: Associations between analysis variables and missing young-adult ADHD/ASD data | | | | | | |
| --- | --- | --- | --- | --- | --- | --- |
|  | Available exposure data for included participants^**^ | | Number of young-adult ADHD/ASD measures available^*^ | | | Association between exposures and missing ASD/ADHD data |
|  |  |  | None (N=8628)^a^ | Partial (N=3560)^b^ | Complete (N=2504)^c^ |  |
|  | % | (N) | Exposure mean (SD) | | | (none vs partial/complete) |
| Male sex | 100% | (6064) | 0.57 (0.49) | 0.47 (0.50) | 0.35 (0.48) | OR=1.82, 95% CI=1.70-1.94 |
| IQ | 76% | (4586) | 99.36 (15.99) | 104.80 (16.27) | 109.04 (15.86) | OR=0.97, 95% CI=0.97-0.98 |
| Reading | 79% | (4796) | 7.00 (2.72) | 7.66 (2.38) | 8.00 (2.12) | OR=0.88, 95% CI=0.86-0.90 |
| Spelling | 79% | (4793) | 9.48 (3.75) | 10.34 (3.33) | 10.94 (3.14) | OR=0.91, 95% CI=0.90-0.92 |
| Pragmatic language | 81% | (4908) | 149.26 (8.88) | 150.93 (7.75) | 151.93 (6.93) | OR=0.97, 95% CI=0.96-0.97 |
| Communication | 71% | (4303) | 166.75 (2.22) | 158.05 (17.76) | 160.21 (15.17) | OR=1.11, 95% CI=0.88-1.41 |
| ADHD PRS | 69% | (4214) | 0.08 (1.00) | -0.05 (1.00) | -0.12 (0.99) | OR=1.18, 95% CI=1.13-1.23 |
| ASD PRS | 69% | (4214) | -0.00 (0.99) | -0.01 (1.03) | 0.02 (1.00) | OR=1.00, 95% CI=0.95-1.04 |
| Schizophrenia PRS | 69% | (4214) | 0.08 (0.99) | -0.01 (1.01) | -0.15 (0.98) | OR=1.17, 95% CI=1.12-1.22 |
| Depression PRS | 69% | (4214) | 0.06 (1.00) | -0.04 (1.01) | -0.08 (0.98) | OR=1.12, 95% CI=1.07-1.17 |
| Anxiety PRS | 69%6 | (4214) | 0.03 (0.98) | -0.03 (1.01) | -0.02 (1.03) | OR=1.06, 95% CI=1.01-1.10 |
| ^*^Out of four primary measures available assessing parent- and self-rated ADHD and ASD symptoms in young-adulthood. ^**^Those with ADHD/ASD measures available in young-adulthood (partial or complete). ^a^0 measures available, ^b^1-3 measures available, ^c^4 measures available. | | | | | | |

| **Supplementary Table 8.** Associations with parent-rated ADHD symptoms in young adulthood using different approached to missing data | | | | | | | | | | | | | | | | |
| --- | --- | --- | --- | --- | --- | --- | --- | --- | --- | --- | --- | --- | --- | --- | --- | --- |
|  |  | Original estimate | | |  | MI: full sample | | |  | IPW: no MI | | |  | Complete cases | | |
|  |  | β | (95% CI) | p |  | β | (95% CI) | p |  | β | (95% CI) | p |  | β | (95% CI) | p |
| IQ |  | -0.24 | (-0.28, -0.20) | 3x10^-30^ |  | -0.27 | (-0.30, -0.23) | 2x10^-33^ |  | -0.21 | (-0.25, -0.18) | 6x10^-26^ |  | -0.21 | (-0.24, -0.18) | 3x10^-37^ |
| Reading |  | -0.22 | (-0.26, -0.18) | 3x10^-26^ |  | -0.26 | (-0.30, -0.22) | 3x10^-28^ |  | -0.21 | (-0.25, -0.17) | 8x10^-23^ |  | -0.20 | (-0.23, -0.17) | 4x10^-36^ |
| Spelling |  | -0.24 | (-0.27, -0.20) | 2x10^-30^ |  | -0.27 | (-0.32 -0.23) | 1x10^-31^ |  | -0.23 | (-0.27, -0.19) | 2x10^-29^ |  | -0.22 | (-0.25, -0.18) | 7x10^-41^ |
| Pragmatic language |  | -0.38 | (-0.42, -0.34) | 1x10^-64^ |  | -0.42 | (-0.46, -0.38 | 2x10^-67^ |  | -0.37 | (-0.42, -0.31) | 2x10^-41^ |  | -0.36 | (-0.39, -0.34) | 4x10^-124^ |
| Communication |  | -0.48 | (-0.54, -0.42) | 3x10^-53^ |  | -0.52 | (-0.56, -0.47) | 2x10^-81^ |  | -0.47 | (-0.55, -0.40) | 4x10^-31^ |  | -0.49 | (-0.51, -0.46) | 1x10^-255^ |
| ADHD PRS |  | 0.12 | (0.08, 0.16) | 7x10^-08^ |  | 0.13 | (0.09, 0.17) | 3x10^-09^ |  | 0.13 | (0.08, 0.17) | 2x10^-08^ |  | 0.10 | (0.07, 0.14) | 1x10^-08^ |
| ASD PRS |  | -0.03 | (-0.08, 0.01) | 0.14 |  | -0.03 | (-0.07, 0.01) | 0.14 |  | -0.03 | (-0.07, 0.01) | 0.16 |  | -0.02 | (-0.06, 0.01) | 0.20 |
| Schizophrenia PRS |  | 0.05 | (0.01, 0.09) | 0.02 |  | 0.06 | (0.02, 0.10) | 5x10^-3^ |  | 0.04 | (0.00, 0.09) | 0.04 |  | 0.03 | (-0.00, 0.07) | 0.05 |
| Depression PRS |  | 0.01 | (-0.03, 0.06) | 0.56 |  | 0.04 | (-0.01, 0.08) | 0.10 |  | 0.01 | (-0.03, 0.05) | 0.75 |  | 0.00 | (-0.04, 0.04) | 1.00 |
| Anxiety PRS |  | 0.01 | (-0.04, 0.05) | 0.78 |  | -0.00 | (-0.04, 0.04) | 0.88 |  | 0.01 | (-0.03, 0.05) | 0.68 |  | 0.01 | (-0.03, 0.04) | 0.60 |
| PRS = polygenic risks score. MI = multiple imputation. IPW = inverse probability weighting. Multivariable analyses for PRS. | | | | | | | | | | | | | | | | |

| **Supplementary Table 9.** Associations with parent-rated ASD symptoms in young adulthood using different approached to missing data | | | | | | | | | | | | | | | | |
| --- | --- | --- | --- | --- | --- | --- | --- | --- | --- | --- | --- | --- | --- | --- | --- | --- |
|  |  | Original estimate | | |  | MI: full sample | | |  | IPW: no MI | | |  | Complete cases | | |
|  |  | β | (95% CI) | p |  | β | (95% CI) | p |  | β | (95% CI) | p |  | β | (95% CI) | p |
| IQ |  | -0.20 | (-0.25, -0.16) | 2x10^-19^ |  | -0.23 | (-0.27, -0.19) | 2x10^-25^ |  | -0.19 | (-0.24, -0.14) | 2x10^-15^ |  | -0.18 | (-0.21, -0.15) | 2x10^-27^ |
| Reading |  | -0.19 | (-0.23, -0.14) | 2x10^-16^ |  | -0.22 | (-0.26, -0.17) | 2x10^-19^ |  | -0.17 | (-0.22 -0.12) | 7x10^-12^ |  | -0.16 | (-0.19 -0.13) | 3x10^-23^ |
| Spelling |  | -0.19 | (-0.23, -0.15) | 3x10^-17^ |  | -0.22 | (-0.27, -0.18) | 1x10^-20^ |  | -0.18 | (-0.22, -0.13) | 1x10^-13^ |  | -0.17 | (-0.20, -0.13) | 2x10^-24^ |
| Pragmatic language |  | -0.38 | (-0.43, -0.34) | 2x10^-57^ |  | -0.43 | (-0.47, -0.39) | 9x10^-69^ |  | -0.37 | (-0.43, -0.32) | 2x10^-40^ |  | -0.37 | (-0.40, -0.34) | 7x10^-131^ |
| Communication |  | -0.60 | (-0.66, -0.55) | 1x10^-104^ |  | -0.63 | (-0.66, -0.59) | 2x10^-122^ |  | -0.60 | (-0.67, -0.53) | 5x10^-62^ |  | -0.63 | (-0.65, -0.60) | <5x10^-308^ |
| ADHD PRS |  | 0.10 | (0.05, 0.14) | 2x10^-05^ |  | 0.11 | (0.07, 0.15) | 3x10^-07^ |  | 0.10 | (0.06, 0.14) | 7x10^-06^ |  | 0.09 | (0.04, 0.12) | 2x10^-06^ |
| ASD PRS |  | -0.04 | (-0.08, 0.00) | 0.07 |  | -0.04 | (-0.09, 0.00) | 0.05 |  | -0.04 | (-0.08, 0.00) | 0.05 |  | -0.03 | (-0.06, 0.01) | 0.16 |
| Schizophrenia PRS |  | 0.06 | (0.02, 0.11) | 0.01 |  | 0.07 | (0.03, 0.11) | 2x10^-03^ |  | 0.06 | (0.02, 0.11) | 0.01 |  | 0.05 | (0.01, 0.08) | 0.01 |
| Depression PRS |  | 0.05 | (0.00, 0.09) | 0.04 |  | 0.06 | (0.02, 0.11) | 2x10^-03^ |  | 0.04 | (-0.00, 0.08) | 0.07 |  | 0.03 | (-0.01, 0.06) | 0.10 |
| Anxiety PRS |  | 0.02 | (-0.03, 0.06) | 0.45 |  | 0.02 | (-0.02, 0.06) | 0.44 |  | 0.01 | (-0.04, 0.06) | 0.73 |  | 0.02 | (-0.02, 0.05) | 0.34 |
| PRS = polygenic risks score. MI = multiple imputation. IPW = inverse probability weighting. Multivariable analyses for PRS only. | | | | | | | | | | | | | | | | |

| **Supplementary Table 10.** Associations with self-rated ADHD symptoms in young adulthood using different approached to missing data | | | | | | | | | | | | | | | | |
| --- | --- | --- | --- | --- | --- | --- | --- | --- | --- | --- | --- | --- | --- | --- | --- | --- |
|  |  | Original estimate | | |  | MI: full sample | | |  | IPW: no MI | | |  | Complete cases | | |
|  |  | β | (95% CI) | p |  | β | (95% CI) | p |  | β | (95% CI) | p |  | β | (95% CI) | p |
| IQ |  | -0.08 | (-0.12, -0.04) | 2x10^-04^ |  | -0.09 | (-0.13, -0.05) | 3x10^-05^ |  | -0.06 | (-0.11, -0.01) | 0.02 |  | -0.04 | (-0.08, -0.01) | 0.02 |
| Reading |  | -0.09 | (-0.13, -0.05) | 2x10^-05^ |  | -0.11 | (-0.15, -0.06) | 5x10^-06^ |  | -0.07 | (-0.11, -0.03) | 2x10^-03^ |  | -0.07 | (-0.11, -0.04) | 3x10^-05^ |
| Spelling |  | -0.11 | (-0.16, -0.07) | 8x10^-08^ |  | -0.13 | (-0.18, -0.08) | 2x10^-08^ |  | -0.09 | (-0.13, -0.04) | 1x10^-04^ |  | -0.09 | (-0.13, -0.06) | 7x10^-08^ |
| Pragmatic language |  | -0.17 | (-0.22, -0.13) | 6x10^-15^ |  | -0.18 | (-0.23, -0.14) | 2x10^-14^ |  | -0.13 | (-0.17, -0.10) | 5x10^-14^ |  | -0.14 | (-0.18, -0.11) | 4x10^-17^ |
| Communication |  | -0.17 | (-0.22, -0.12) | 8x10^-12^ |  | -0.19 | (-0.24, -0.14) | 1x10^-12^ |  | -0.14 | (-0.19, -0.10) | 4x10^-11^ |  | -0.15 | (-0.19, -0.11) | 2x10^-14^ |
| ADHD PRS |  | 0.08 | (0.03, 0.12) | 5x10^-04^ |  | 0.08 | (0.04, 0.12) | 1x10^-04^ |  | 0.06 | (0.02, 0.10) | 7x10^-03^ |  | 0.06 | (0.03, 0.10) | 1x10^-03^ |
| ASD PRS |  | -0.05 | (-0.10, -0.01) | 0.01 |  | -0.05 | (-0.09, -0.01) | 0.02 |  | -0.05 | (-0.09, -0.01) | 0.01 |  | -0.05 | (-0.09, -0.01) | 0.01 |
| Schizophrenia PRS |  | 0.02 | (-0.02, 0.06) | 0.41 |  | 0.03 | (-0.02, 0.07) | 0.21 |  | 0.01 | (-0.03, 0.05) | 0.78 |  | 0.02 | (-0.02, 0.05) | 0.41 |
| Depression PRS |  | 0.07 | (0.02, 0.11) | 3x10^-03^ |  | 0.07 | (0.02, 0.11) | 2x10^-03^ |  | 0.08 | (0.04, 0.12) | 2x10^-04^ |  | 0.06 | (0.02, 0.10) | 2x10^-03^ |
| Anxiety PRS |  | 0.05 | (0.01, 0.09) | 0.02 |  | 0.04 | (-0.00, 0.08) | 0.05 |  | 0.05 | (0.01, 0.09) | 8x10^-03^ |  | 0.04 | (0.00, 0.07) | 0.04 |
| PRS = polygenic risks score. MI = multiple imputation. IPW = inverse probability weighting. Multivariable analyses for PRS. | | | | | | | | | | | | | | | | |

| **Supplementary Table 11.** Associations with self-rated ASD symptoms in young adulthood using different approached to missing data | | | | | | | | | | | | | | | | |
| --- | --- | --- | --- | --- | --- | --- | --- | --- | --- | --- | --- | --- | --- | --- | --- | --- |
|  |  | Original estimate | | |  | MI: full sample | | |  | IPW: no MI | | |  | Complete cases | | |
|  |  | β | (95% CI) | p |  | β | (95% CI) | p |  | β | (95% CI) | p |  | β | (95% CI) | p |
| IQ |  | -0.14 | (-0.18, -0.10) | 5x10^-12^ |  | -0.16 | (-0.21, -0.12) | 5x10^-13^ |  | -0.12 | (-0.16, -0.07) | 8x10^-08^ |  | -0.10 | (-0.14, -0.07) | 2x10^-08^ |
| Reading |  | -0.12 | (-0.16, -0.08) | 1x10^-09^ |  | -0.14 | (-0.19, -0.10) | 1x10^-08^ |  | -0.08 | (-0.12, -0.04) | 1x10^-04^ |  | -0.09 | (-0.12, -0.05) | 6x10^-07^ |
| Spelling |  | -0.12 | (-0.16, -0.08) | 7x10^-10^ |  | -0.15 | (-0.19, -0.10) | 3x10^-09^ |  | -0.08 | (-0.12, -0.05) | 2x10^-05^ |  | -0.08 | (-0.12, -0.05) | 2x10^-06^ |
| Pragmatic language |  | -0.23 | (-0.27, -0.19) | 5x10^-27^ |  | -0.25 | (-0.30, -0.21) | 1x10^-23^ |  | -0.20 | (-0.24, -0.16) | 1x10^-22^ |  | -0.21 | (-0.24, -0.18) | 4x10^-33^ |
| Communication |  | -0.28 | (-0.32, -0.23) | 5x10^-30^ |  | -0.29 | (-0.34, -0.24) | 6x10^-25^ |  | -0.25 | (-0.30, -0.20) | 7x10^-25^ |  | -0.26 | (-0.30, -0.22) | 3x10^-39^ |
| ADHD PRS |  | 0.03 | (-0.01, 0.07) | 0.20 |  | 0.04 | (-0.01, 0.08) | 0.09 |  | 0.01 | (-0.03, 0.05) | 0.62 |  | 0.01 | (-0.03, 0.05) | 0.57 |
| ASD PRS |  | -0.00 | (-0.05, 0.04) | 0.92 |  | -0.00 | (-0.04, 0.04) | 0.97 |  | 0.00 | (-0.04, 0.05) | 0.82 |  | 0.00 | (-0.03, 0.04) | 0.88 |
| Schizophrenia PRS |  | 0.04 | (0.00, 0.08) | 0.05 |  | 0.05 | (0.01, 0.09) | 0.03 |  | 0.03 | (-0.01, 0.08) | 0.16 |  | 0.03 | (-0.00, 0.07) | 0.07 |
| Depression PRS |  | 0.05 | (0.00, 0.09) | 0.04 |  | 0.05 | (0.01, 0.09) | 0.02 |  | 0.06 | (0.02, 0.10) | 2x10^-03^ |  | 0.03 | (-0.00, 0.07) | 0.08 |
| Anxiety PRS |  | 0.04 | (-0.01, 0.08) | 0.10 |  | 0.03 | (-0.01, 0.07) | 0.14 |  | 0.04 | (-0.01, 0.08) | 0.09 |  | 0.03 | (-0.00, 0.07) | 0.08 |
| PRS = polygenic risks score. MI = multiple imputation. IPW = inverse probability weighting. Multivariable analyses for PRS only. | | | | | | | | | | | | | | | | |

| **Supplementary Table 12.** Associations between variables include in the inverse probability weight and missing young-adult ADHD/ASD data | | |
| --- | --- | --- |
|  | Initial multivariable analyses | Final multivariable analysis for IPW |
| Male sex | OR=1.93, 95% CI=1.80-2.08 | OR=1.93, 95% CI=1.80-2.07 |
| Social disadvantage | OR=1.58, 95% CI=1.43-1.75 | OR=1.58, 95% CI=1.43-1.75 |
| Maternal age at birth | OR=0.93, 95% CI=0.92-0.94 | OR=0.93, 95% CI=0.92-0.94 |
| Maternal education | OR=0.70, 95% CI=0.68-0.72 | OR=0.70, 95% CI=0.68-0.72 |
| Low birth weight | OR=1.29, 95% CI=1.05-1.58 | OR=1.27, 95% CI=1.07-1.51 |
| Preterm birth | OR=0.97, 95% CI=0.80-1.19 | - |
| Smoking in pregnancy | OR=1.12, 95% CI=1.01-1.25 | OR=1.12, 95% CI=1.01-1.25 |
| Maternal depression | OR=1.20, 95% CI=1.04-1.38 | OR=1.20, 95% CI=1.04-1.37 |
| Parity | OR=1.12, 95% CI=1.08-1.17 | OR=1.12, 95% CI=1.08-1.17 |

| **Supplementary Table 13.** Additional measures of ADHD/ASD included in the multiple imputation model: correlations with young-adult ADHD/ASD measures | | | | | | |
| --- | --- | --- | --- | --- | --- | --- |
|  | Available exposure data for included participants^**^ | | Correlation with young-adult ADHD/ASD | | | |
|  |  |  | Parent-rated | | Self-rated | |
|  | % | (N) | ADHD | ASD | ADHD | ASD |
| Parent-rated ADHD |  |  |  |  |  |  |
| Age 4 years SDQ | 83% | (5058) | 0.30 | 0.19 | 0.15 | 0.12 |
| Age 7 years SDQ | 80% | (4855) | 0.38 | 0.23 | 0.20 | 0.13 |
| Age 8 years SDQ | 79% | (4796) | 0.43 | 0.27 | 0.23 | 0.13 |
| Age 10 years SDQ | 82% | (4979) | 0.44 | 0.29 | 0.23 | 0.15 |
| Age 12 years SDQ | 80% | (4842) | 0.48 | 0.30 | 0.26 | 0.17 |
| Age 13 years SDQ | 78% | (4750) | 0.49 | 0.30 | 0.28 | 0.16 |
| Age 17 years SDQ | 70% | (4270) | 0.52 | 0.32 | 0.29 | 0.14 |
| Parent-rated ASD |  |  |  |  |  |  |
| Age 7 years SCDC | 79% | (4806) | 0.34 | 0.37 | 0.14 | 0.15 |
| Age 10 years SCDC | 81% | (4900) | 0.37 | 0.43 | 0.16 | 0.16 |
| Age 13 years SCDC | 79% | (4769) | 0.40 | 0.50 | 0.18 | 0.18 |
| Age 17 years SCDC | 70% | (4273) | 0.38 | 0.48 | 0.15 | 0.14 |
| Age 25 years AQ | 72% | (4377) | 0.45 | 0.59 | 0.17 | 0.45 |
| ^**^Those with ADHD/ASD measures available in young-adulthood (partial or complete). SDQ=Strengths and Difficulties Questionnaire, SCDC=Social Communication Disorders Checklist, AQ=Autism Spectrum Quotient. | | | | | | |

**References**

Boyd, A., Golding, J., Macleod, J., Lawlor, D. A., Fraser, A., Henderson, J., . . . Davey Smith, G. (2013). Cohort Profile: the 'children of the 90s'--the index offspring of the Avon Longitudinal Study of Parents and Children. *Int J Epidemiol, 42*(1), 111-127.

Cooper, M., Hammerton, G., Collishaw, S., Langley, K., Thapar, A., Dalsgaard, S., . . . Riglin, L. (2018). Investigating late-onset ADHD: a population cohort investigation. *Journal of child psychology and psychiatry, and allied disciplines, 59*(10), 1105-1113.

Demontis, D., Walters, R. K., Martin, J., Mattheisen, M., Als, T. D., Agerbo, E., . . . Neale, B. M. (2019). Discovery of the first genome-wide significant risk loci for attention deficit/hyperactivity disorder. *Nat Genet, 51*(1), 63-75.

Euesden, J., Lewis, C. M., & O'Reilly, P. F. (2015). PRSice: Polygenic Risk Score software. *Bioinformatics, 31*(9), 1466-1468.

Fraser, A., Macdonald-Wallis, C., Tilling, K., Boyd, A., Golding, J., Davey Smith, G., . . . Lawlor, D. A. (2013). Cohort Profile: the Avon Longitudinal Study of Parents and Children: ALSPAC mothers cohort. *Int J Epidemiol, 42*(1), 97-110.

Grove, J., Ripke, S., Als, T. D., Mattheisen, M., Walters, R. K., Won, H., . . . Borglum, A. D. (2019). Identification of common genetic risk variants for autism spectrum disorder. *Nat Genet, 51*(3), 431-444.

Hyde, C. L., Nagle, M. W., Tian, C., Chen, X., Paciga, S. A., Wendland, J. R., . . . Winslow, A. R. (2016). Identification of 15 genetic loci associated with risk of major depression in individuals of European descent. *Nat Genet, 48*(9), 1031-1036.

Northstone, K., Lewcock, M., Groom, A., Boyd, A., Macleod, J., Timpson, N., & Wells, N. (2019). The Avon Longitudinal Study of Parents and Children (ALSPAC): an update on the enrolled sample of index children in 2019. *Wellcome open research, 4*, 51-51.

Pardinas, A. F., Holmans, P., Pocklington, A. J., Escott-Price, V., Ripke, S., Carrera, N., . . . Walters, J. T. R. (2018). Common schizophrenia alleles are enriched in mutation-intolerant genes and in regions under strong background selection. *Nat Genet, 50*(3), 381-389.

Purves, K. L., Coleman, J. R. I., Meier, S. M., Rayner, C., Davis, K. A. S., Cheesman, R., . . . Eley, T. C. (2019). A major role for common genetic variation in anxiety disorders. *Molecular Psychitary, doi:10.1038/s41380-019-0559-1*.

Schizophrenia Working Group of the Psychiatric Genomics Consortium. (2014). Biological insights from 108 schizophrenia-associated genetic loci. *Nature, 511*(7510), 421-427.

Seaman, S. R., White, I. R., Copas, A. J., & Li, L. (2012). Combining multiple imputation and inverse-probability weighting. *Biometrics, 68*(1), 129-137.

von Hippel, P. T. (2018). How Many Imputations Do You Need? A Two-stage Calculation Using a Quadratic Rule. *Sociological Methods & Research*, doi: 10.1177/0049124117747303.

White, I. R., Royston, P., & Wood, A. M. (2011a). Multiple imputation using chained equations: issues and guidance for practice. *Statistics in medicine, 30*(4), 377-399.

White, I. R., Royston, P., & Wood, A. M. (2011b). Multiple imputation using chained equations: Issues and guidance for practice. *Stat Med, 30*(4), 377-399.

Wray, N. R., Ripke, S., Mattheisen, M., Trzaskowski, M., Byrne, E. M., Abdellaoui, A., . . . Major Depressive Disorder Working Group of the Psychiatric Genomics, C. (2018). Genome-wide association analyses identify 44 risk variants and refine the genetic architecture of major depression. *Nat Genet, 50*(5), 668-681.
